# Postsynaptic insertion of AMPA receptor onto cortical pyramidal neurons in the anterior cingulate cortex after peripheral nerve injury

**DOI:** 10.1186/s13041-014-0076-8

**Published:** 2014-10-31

**Authors:** Tao Chen, Wen Wang, Yu-Lin Dong, Ming-Ming Zhang, Jian Wang, Kohei Koga, Yong-Hui Liao, Jin-Lian Li, Timotheus Budisantoso, Ryuichi Shigemoto, Makoto Itakura, Richard L Huganir, Yun-Qing Li, Min Zhuo

**Affiliations:** Center for Neuron and Disease, Frontier Institute of Science and Technology, Xi’an Jiaotong University, Xi’an, China; Department of Anatomy, Histology and Embryology and K.K. Leung Brain Research Center, the Fourth Military Medical University, Xi’an, 710032 China; Department of Physiology, Faculty of Medicine, Center for the Study of Pain, University of Toronto, 1 King’s College Circle, Toronto, Ontario M5S 1A8 Canada; Division of Cerebral Structure, National Institute for Physiological Sciences, Myodaiji, Okazaki 444-8787 Japan; Department of Biochemistry, Kitasato University School of Medicine, Sagamihara, Kanagawa, Japan; Department of Neuroscience and Howard Hughes Medical Institute, Johns Hopkins University School of Medicine, Baltimore, MD USA

## Abstract

Long-term potentiation (LTP) is the key cellular mechanism for physiological learning and pathological chronic pain. Postsynaptic accumulation of AMPA receptor (AMPAR) GluA1 plays an important role for injury-related cortical LTP. However, there is no direct evidence for postsynaptic GluA1 insertion or accumulation after peripheral injury. Here we report nerve injury increased the postsynaptic expression of AMPAR GluA1 in pyramidal neurons in the layer V of the anterior cingulate cortex (ACC), including the corticospinal projecting neurons. Electrophysiological recordings show that potentiation of postsynaptic responses was reversed by Ca^2+^ permeable AMPAR antagonist NASPM. Finally, behavioral studies show that microinjection of NASPM into the ACC inhibited behavioral sensitization caused by nerve injury. Our findings provide direct evidence that peripheral nerve injury induces postsynaptic GluA1 accumulation in cingulate cortical neurons, and inhibits postsynaptic GluA1 accumulation which may serve as a novel target for treating neuropathic pain.

## Introduction

Glutamate is the major excitatory transmitter in the central nervous system, from the spinal cord to cortex [1,2]. Most of the postsynaptic currents are mediated by AMPA receptors, while NMDA receptor and metabotropic glutamate receptors mainly contribute to the induction and regulation of synaptic plasticity - including long term potentiation (LTP) and long term depression (LTD) [3-6]. Hippocampal synapses are mostly investigated as a result of its important role in learning and memory. Depending on the induction protocols, recording methods and central regions, there are two major forms of synaptic mechanisms - presynaptic enhancement of glutamate release, and/or postsynaptic potentiation of AMPARs –which contribute to hippocampal potentiation [3,4,6,7]. Recent studies in adult cortical areas indicate that similar LTP takes place in adult cortical excitatory synapses [2,8,9]. Under pathological conditions such as peripheral chronic inflammation or nerve injury, excitatory synaptic transmission in the ACC is potentiated for a long term period of time [8-13]. Integrative experimental approaches including electrophysiological, pharmacological and biochemical methods demonstrate that AMPA GluA1 receptors are required for injury induced potentiation [11,14-17].

The subunits of AMPAR (GluA1-GluA4) are dynamic at synaptic sites and their trafficking onto plasma membrane plays an important role in synaptic potentiation such as LTP [3,5,18]. However, previous evidence that links postsynaptic AMPAR trafficking to behavioral learning is mostly indirect in mammals. Evidence for experience strengthened synaptic transmission in the central synapses came from electrophysiological and pharmacological experiments [19,20]. A recent study in the snail sensory ganglia reported learning-induced AMPAR trafficking [21]. To our knowledge, there is no direct evidence for learning or experience driven AMPAR trafficking at central synapses in behavioral animals. In the present study, we demonstrate that nerve injury induced accumulation of AMPAR GluA1 in postsynaptic membranes of the ACC pyramidal neurons in adult mice. Furthermore, we show that GluA1 accumulation occurred exclusively in the corticospinal projecting cells, highlighting its important roles in sensory modulation at the level of the spinal cord. Using GluA1 phosphorylation site knock-in mice, we find that protein kinase A (PKA) phosphorylation of GluA1was critical for the nerve injury induced potentiation and behavioral sensitization. Finally, we show that application of Ca^2+^ permeable AMPAR antagonist NAPSM reduced LTP in the ACC, as well as behavioral sensitization in freely moving mice.

## Results

### Nerve injury causes long-term increases in postsynaptic GluA1 subunits

**Figure 1:**
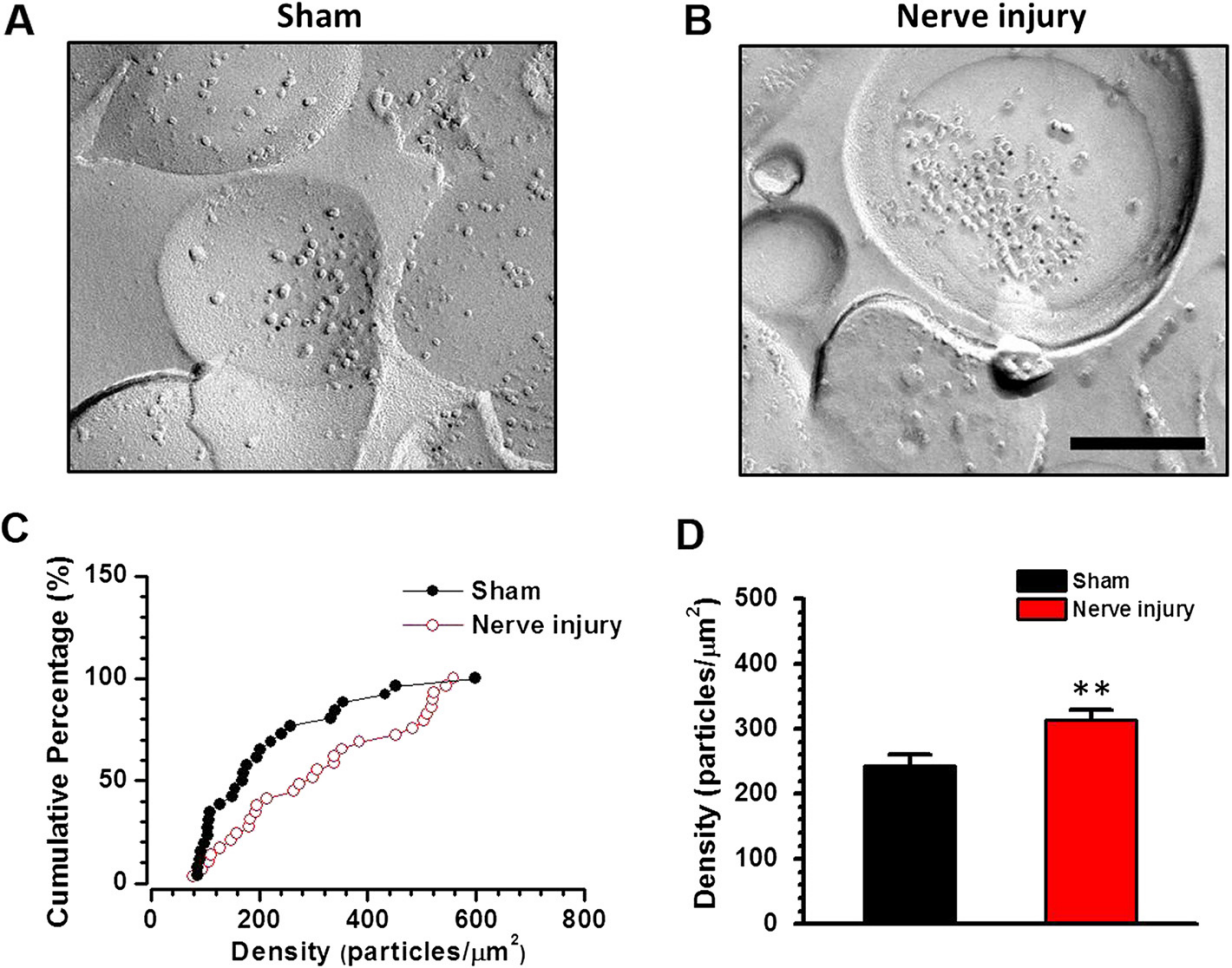
**Postsynaptic accumulation of GluA1 in the ACC after nerve injury. (A-B)** SDS-digested freeze fracture replica labeling EM samples showing that increased GluA1 particles in the synaptic region on ACC layer V neurons in mice with nerve injury compared with mice with sham surgery. Bar equals to 200 nm. **(C)** Cumulative histograms showing the distribution of postsynaptic GluA1 density. **(D)** Averaged density of the postsynaptic GluA1. **, *p* <0.01.

AMPAR GluA1 subunit trafficking into the postsynaptic region is believed to be important for synaptic potentiation [22-26]. By applying whole-cell patch-clamp recording, we have confirmed that nerve injury potentiates AMPAR mediated evoked EPSCs (eEPSCs) in the pyramidal cells in layer V of the ACC [15]. We wanted to check whether the potentiated electrophysiological responses were due to increased expression of postsynaptic GluA1. SDS digested freeze-fracture replica labeling (SDS-FRL) method was employed, in which the two-dimensional cell membrane structure, as well as the distribution and density of GluA1 could be examined at individual synapses of the ACC layer V neurons [27]. The excitatory postsynaptic area was identified with the clustering of intra-membrane particles on the exoplasmic fracture face (E-face). In the ACC layer V from both nerve injured and sham operated mice, immunogold particles for GluA1 on the E-face were detected in postsynaptic sites (Figure 1A-B). We found that the distribution of synaptic GluA1 density from the nerve injured mouse was right-shifted compared with that from the sham operated mouse (Figure 1C). The mean GluA1 density for the nerve injured group (313.2 ± 16.0 particles/μm^2^, n = 89 synapses/3 mice) was significantly larger than that for the sham-operated group (243.7 ± 16.4 particles/μm^2^, n = 86 synapses/3 mice; *p* <0.01) (Figure 1D). These results indicate that GluA1 is recruited to the postsynaptic area in layer V after nerve injury.

### Accumulation of postsynaptic GluA1 on spinal cord projecting neurons in the ACC

**Figure 2:**
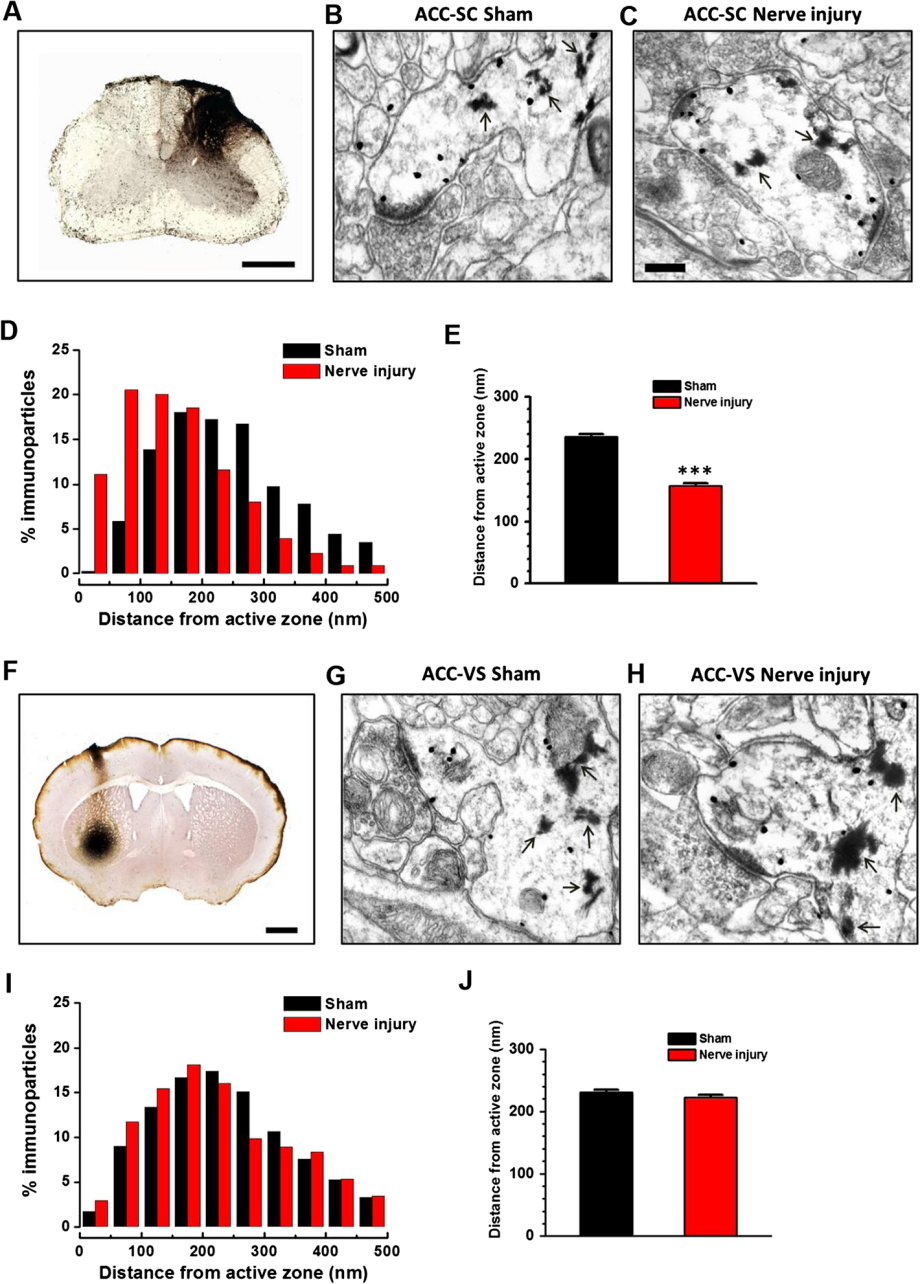
**Nerve injury accumulated postsynaptic GluA1 in ACC**-**SC projecting neurons. (A-C)** Samples showing that immune-gold particles labeled GluA1 were distributed within the HRP-labeled dendrite and spine with HRP injection into the dorsal part of the SC **(A)**. **(D)** Percentage of GluA1 particles in 50 nm bins as a function of distance from the nearest edge of synapses. **(E)** Averaged distances of GluA1 from the edge of synapses. **(F-J)** Samples and plotted figures showing the distribution of GluA1 within the HRP-labeled dendrite and spine with HRP injection into the VS **(F)**. Bars equal to 1000 μm in **(F)**, 500 μm in **(A)** and 2 μm in **(B, C, G, H)**. ***, *p* <0.001.

In our previous work [15], we found that nerve injury potentiated AMPAR mediated eEPSCs in the layer V of the ACC, and this potentiation also occurred on spinal cord projecting pyramidal cells. We wanted to determine if GluA1 accumulation also happened on spinal cord (SC) projecting neurons in the ACC. To achieve this purpose, we injected horseradish peroxidase (HRP) into one side of the dorsal part of the SC (Figure 2A) and observed the GluA1 immunogold staining in HRP retrograde labeling neurons in the ACC, by exploring pre-embedding immunostaining transmission electron microscopic (EM) method. After HRP injection into the dorsal horn of SC, HRP retrogradely labeled neurons were mainly found in layer V of the ACC. Using the electron microscope we measured the distances between individual gold particles and the closest edge of postsynaptic active zone of asymmetric synapses in HRP labeled dendritic profiles and cell bodies of the layer V neurons. We found that more GluA1 particles were closer to the synaptic active zone in corticospinal projecting cells from the nerve injured animals (157.7 ± 3.4 nm, n = 767 particles/135 synapses/3 mice) than those from the sham operated animals (236.1 ± 3.8 nm, n = 751 particles/164 synapses/3 mice. *p* <0.001) (Figure 2B-E), suggesting GluA1 was accumulated to postsynaptic region of the SC projecting neurons in the ACC. In comparison, we checked whether nerve injury caused GluA1 accumulation in ACC-ventral striatum (VS) projecting neurons, which are more likely to be involved in reward function [28]. After HRP injection into one side of the VS (Figure 2F), we found that the HRP retrograde labeled neurons were also mainly distributed in the layer V of the ACC. However, the distances between GluA1 immunoreactive gold particles and the closest edge of postsynaptic active zone of asymmetric synapses in HRP labeled profiles were not significantly different in mice with nerve injury or sham operation (nerve injury: 222.5 ± 4.1 nm, n = 719 particles/158 synapses/3 mice; sham: 231.1 ± 4.1 nm, n = 737 particles/157 synapses/3 mice. *p* >0.05) (Figure 2G-J). These findings indicate that nerve injury does not enhance postsynaptic GluA1 accumulation in the ACC-VS projecting neurons. In addition, we found that the mean number of postsynaptic GluA1 immunoparticles was significantly higher in the SC projecting neurons from nerve injury group compared with sham operated group (sham surgery: 4.59 ± 0.17, nerve injury: 5.67 ± 0.26, *p* <0.001). However, there is no significant difference in the VS projecting neurons between nerve injury and sham operated group (sham surgery: 4.70 ± 0.15, nerve injury: 4.57 ± 0.18, *p* >0.05). These results strongly suggest that nerve injury induced postsynaptic GluA1 accumulation is selective for SC but not VS projecting neurons in the ACC.

### Peak-scaled nonstationary fluctuation analysis

**Figure 3:**
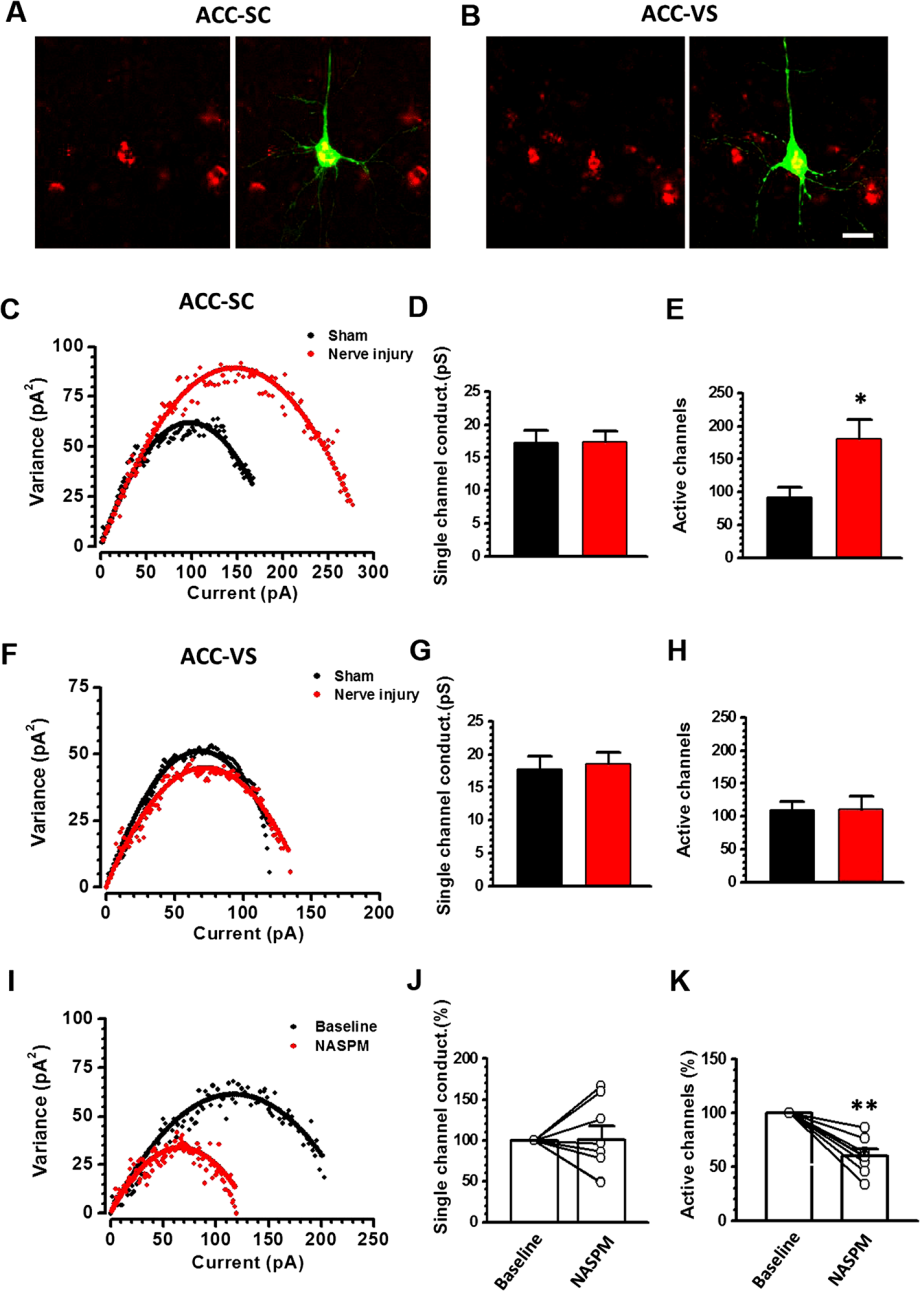
**Nerve injury enhanced the GluA1 channel numbers in ACC**-**SC projecting neurons. (A-B)** Samples showed that DiI retrograde labeling pyramidal cells in the ACC layer V was dual labeled with biocytin for injection into the SC **(A)** or VS **(B)**. Bar equal to 20 μm. **(C-E)** Peak-scaled nonstationary fluctuation analysis (NSFA) indicated that the AMPAR channel number but not the single channel conductance was increased in ACC-SC projecting neurons in mice after nerve injury. **(F-H)** NSFA showed that nerve injury changed neither the AMPAR channel number nor the single channel conductance in ACC-VS projecting neurons. **(I-K)** Bath application of CP-AMPAR antagonist NASPM reduces the AMPAR channel number, without changing the conductance in ACC-SC projecting neurons. *, *p* <0.05; **, *p* <0.01.

Next, we carried out electrophysiological experiments to determine if nerve injury increases GluA1 mediated responses in the layer V of the ACC. The AMPAR mediated eEPSCs on SC or VS projecting neurons in the ACC were recorded and the peak-scaled nonstationary fluctuation analysis (NSFA) was applied to compare their AMPAR unitary conductance (γ) and number of active channels in mice with nerve injury or sham operation [29,30]. After retrograde tracer DiI injection into one side of the SC or VS, retrograde labeling pyramidal cells were recorded using whole- cell patch recording (Figure 3A-B). AMPAR mediated eEPSCs were induced by stimulating local fibers in the layer II/III of the ACC (similar as in [15]). In ACC-SC projecting neurons, we found that the number of active channels (sham surgery: 92.8 ± 17.4, nerve injury: 181.4 ± 29.1, *p* <0.05. n = 10 in each group) but not the unitary conductance (sham surgery: 17.3 ± 2.0 pS, nerve injury: 18.0 ± 1.5 pS, *p* >0.05) was increased by nerve injury (Figure 3C-E). However, in ACC-VS projecting neurons, neither the number of active channels (sham surgery: 109.6 ± 12.6, nerve injury: 111.1 ± 19.2, *p* >0.05. n = 7 in each group) nor the unitary conductance (sham surgery: 17.7 ± 2.0 pS, nerve injury: 18.6 ± 1.7 pS, *p* >0.05) was changed (Figure 3F-H). These results strongly suggest that nerve injury increases the postsynaptic active channels of AMPAR, as well as the AMPAR mediated responses in ACC-SC projecting neurons.

AMPAR is heterotetramer of four homologous subunits (GluA1 to GluA4) that combine in different stoichiometries to form different subunits [25]. GluA2-containing AMPARs are Ca^2+^ impermeable and considered to constitutively traffic to postsynaptic regions. During synaptic potentiation, GluA2 can be replaced by Ca^2+^ permeable AMPAR subunits (CP-AMPAR), which are mainly consist of GluA1 homomers [15,23,31]. We then applied CP-AMPAR antagonist NASPM on ACC-SC projecting neurons from mice with nerve injury to check whether the increased channels are predominately of CP-AMPAR. We found that bath application of NASPM significantly decreased the number of active channels to a mean 60.0 ± 6.3% of the baseline (n =8, *p* <0.01), whereas γ was not affected (101.4 ± 17.3% of baseline, *p* >0.05) (Figure 3I-K). NASPM has no effect on ACC-SC projecting neurons from sham-operated mice (data not shown). These findings are in consistence with the EM results and further support the notion that nerve injury increased the number of postsynaptic GluA1 in ACC-SC projecting neurons.

### Calcium-permeable AMPAR contributes to the LTP in ACC layer V neurons

**Figure 4:**
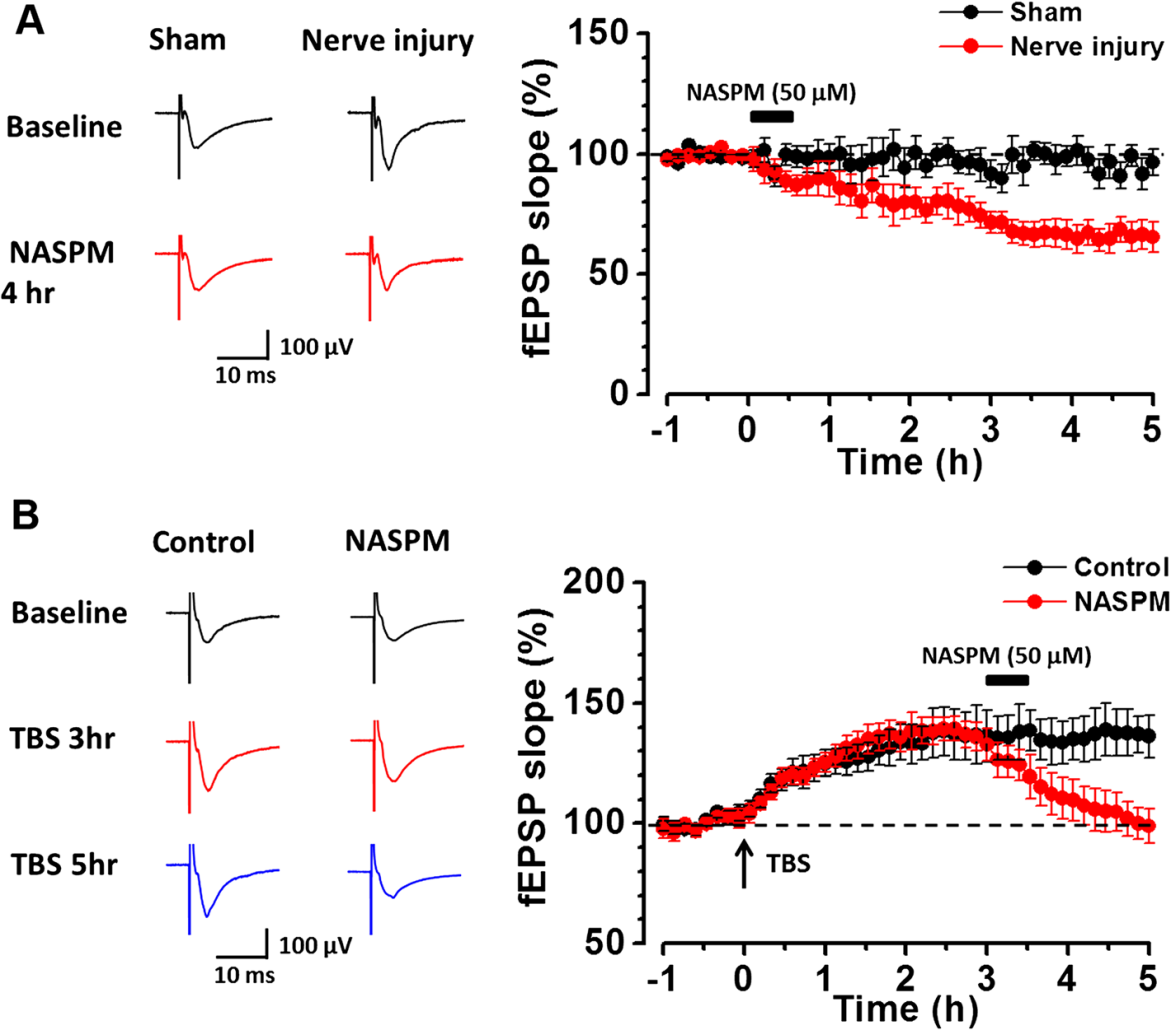
**NASPM had a long term inhibition on the LTP of ACC layer V neurons. (A)** Samples and averaged results showing that bath application of NASPM (50 μM) for 30 min reduced the slope of fEPSP from layer V neurons in mice with nerve injury but not in mice with sham surgery. **(B)** Samples and averaged results showing that TBS induced stable long term potentiation of the fEPSP of layer V neurons. However, bath application of CP-AMPAR antagonist NASPM for 30 min reversed the LTP effect.

From our present and previous results [15], it has been shown that nerve injury induced the postsynaptic accumulation of GluA1 and potentiated synaptic responses of the ACC layer V neurons, including those projecting to the SC. In comparison with mice with sham surgery, a significant inward rectification of the AMPAR mediated *I*-*V* curve were observed in ACC pyramidal cells in mice with nerve injury, indicating that the GluA2 containing AMPAR were replaced by CP-AMPAR [15]. However, it is difficult to determine the time course of NASPM produced inhibition using whole-cell patch recording. We next recorded the field excitatory postsynaptic potentials (fEPSPs) in layer V of the ACC using a Med64 extracellular recording system [32,33]. Similar to our whole-cell patch-clamp recording results [15], the slopes of fEPSPs were significantly larger in mice with nerve injury than that in mice with sham surgery (Figure 4A). Meanwhile, bath application of NASPM for 30 min reduced the potentiated fEPSPs in mice with nerve injury, and the inhibitory effect lasted for at least 5 hrs (66.2 ± 5.4% of the baseline, at 5 hr after NASPM application. *p* <0.01, n = 6 slices). However, NASPM had no effect on the fEPSP recorded from mice with sham surgery (93.6 ± 3.7% of the baseline, at 5 hr after NASPM application. *p* >0.05, n = 6 slices) (Figure 4A).

We then applied theta-burst stimulation (TBS) to induce a LTP in the layer V neurons in naïve mice and found that NASPM reversed the potentiated fEPSP slopes. In control group, TBS induced a long-lasting potentiation of synaptic responses (mean 136.6 ± 10.2% of the baseline at 3 hr; 136.8 ± 8.1% of the baseline at 5 hr; n = 6). In group with NASPM application, TBS induced potentiation was reduced to near baseline level (to 100.8 ± 6.3% of the baseline at 5 hr; n = 6) (Figure 4B). These results demonstrate that the increased CP-AMPAR contributes to the potentiated AMPAR responses.

### Contribution of CP-AMPARs to nerve injury induced mechanical hyperalgesia

**Figure 5:**
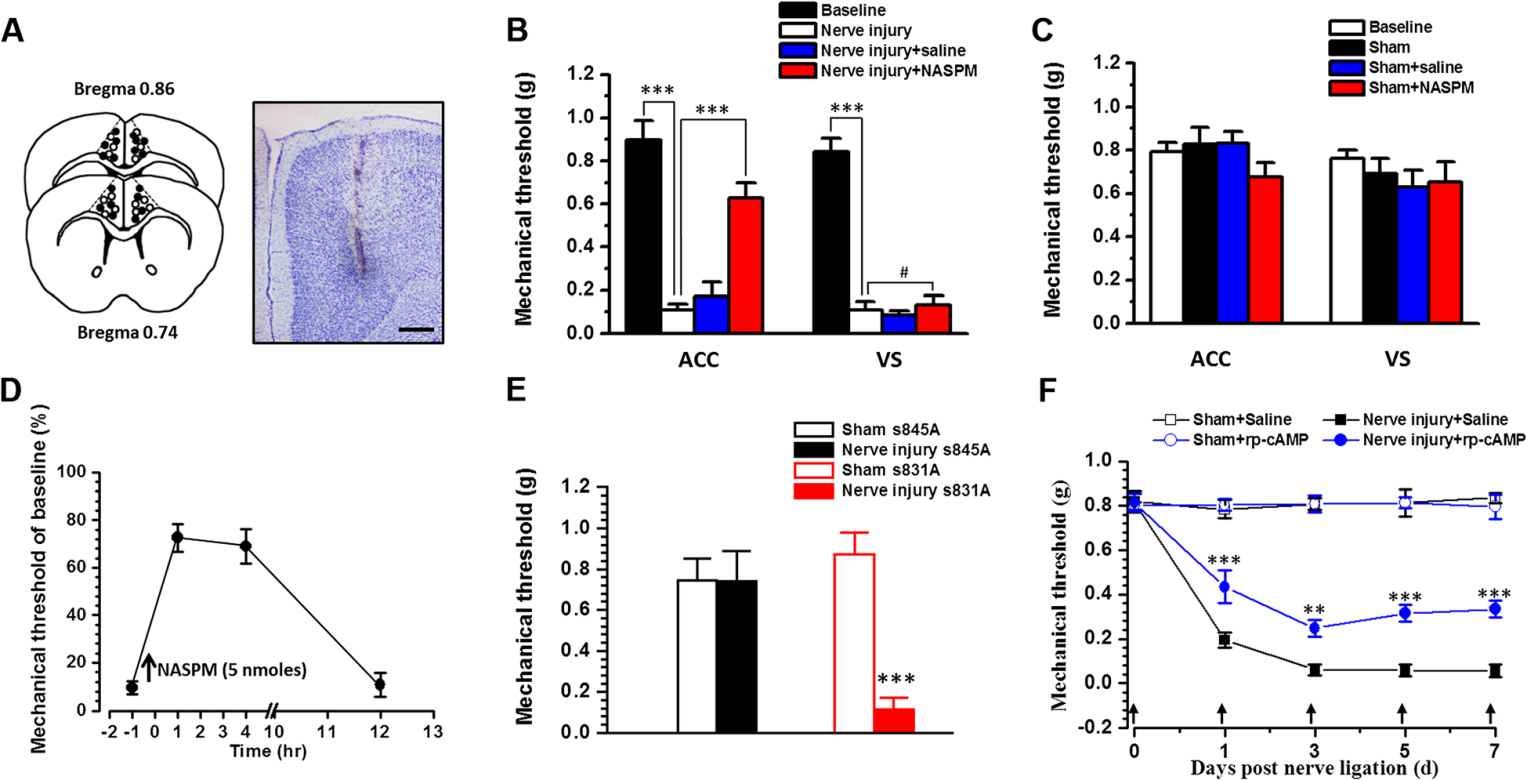
**PKA phosphorylation of GluA1 in the ACC was important for the nerve injury induced mechanical hyperalgesia. (A)** Diagram results showed the location of NASPM (filled circle) or saline (open circle) injection sites in the ACC. Bar equals to 200 μm. **(B)** Nerve injury induced mechanical hypersensitivity in wild type mice, which can be blocked by injection of NASPM into the ACC but not VS. **(C)** NASPM injection into the ACC or VS cannot change the mechanical threshold of mice with sham surgery. **(D)** NASPM’s effect lasts for at least 4 hrs and disappeared 12 hrs after injection into the ACC in mice with nerve injury. **(E)** Summarized behavior results showing that nerve injury induced mechanical hypersensitivity is absent in s845A but not in s831A mice. **(F)** Nerve injury induced mechanical hyperalgesia was inhibited by PKA inhibitor Rp-cAMP consecutive injection into the ACC (before nerve ligation surgery, day 1, day 3, day 5 and day 7 after ligation surgery). ^#^ *p* >0.05, **p <0.01; ***p <0.001.

Does the enhancement and accumulation of CP-AMPAR within the ACC contribute to injury induced mechanical hyperalgesia? To address this question, we injected NASPM into the bilateral ACC (5 nmoles, 0.5 μl per side) in mice at seven days after nerve injury (Figure 5A). We found that nerve injury significantly reduced the mechanical withdrawal threshold of adult mice (n = 16 mice, *p* <0.001), and that microinjections of NASPM into the ACC attenuated mechanical hyperalgesia (n = 9 mice, *p* <0.001) (Figure 5B), which was persisted for at least 4 hr and disappeared 12 hr later (Figure 5D). Meanwhile, NASPM injection into the VS did not affect the mechanical threshold on mice with nerve injury (n = 7 mice, *p* >0.05) (Figure 5B), indicating that the ACC is specific for NASPM’s analgesic effect in neuropathic pain from adult mice. Finally, NASPM injection into the ACC or VS did not affect the mechanical threshold in mice with sham surgery (*p* >0.05) (Figure 5C).

### GluA1 PKA phosphorylation contributed to the mechanical hyperalgesia

Phosphorylation of GluA1 is important for GluA1 trafficking and synaptic plasticity [24,34]. Previous studies have shown that nerve injury induced potentiation of ACC layer V neurons could be blocked in mice with an GluA1 PKA phosphorylation site serine-845 mutation (s845A) but not in mice with GluA1 PKC phosphorylation site serine-831 mutation (s831A) [15]. However, it is unknown if PKA and/or PKC phosphorylation of GluA1 is required for nerve injury induced mechanical hyperalgesia. Taking advantage of genetically induced GluA1 phosphorylation site knockin mice [23], we tested whether GluA1 phosphorylation was necessary. We found that nerve injury induced obvious mechanical hyperalgesia in s831A mice (baseline: 0.87 ± 0.11 g, nerve injury: 0.11 ± 0.06 g, n = 6 mice, *p* <0.001) (Figure 5E). In contrast, in s845A mice, nerve injury induced mechanical hyperalgesia were completely blocked (baseline: 0.74 ± 0.10 g, nerve injury: 0.74 ± 0.14 g, n = 7 mice, *p* >0.05) (Figure 5E). These results suggest that PKA phosphorylation site serine-845 of AMPAR GluA1 plays important roles in neuropathic pain. Since genetic manipulation is not specific to the ACC region, we carried out additional pharmacological experiments to test the role of PKA in the ACC. The microinjection of a PKA inhibitor Rp-cAMP (1 nmoles, 0.5 μl per side) into the ACC produced analgesic effects in mice with nerve injury (Figure 5F), supporting the important role of ACC PKA activity in neuropathic pain.

## Discussion

In this study, we provide direct evidence that AMPAR GluA1 subunit is inserted into postsynaptic sites of the layer V pyramidal cells after peripheral nerve injury. The newly inserted GluA1 receptors contribute to injury-induced synaptic LTP in the ACC and behavioral sensi-tization. There are at least two different mechanisms for ACC’s contribution to chronic pain. Neurons in the ACC may play important roles in pain perception [2,8,35], and enhanced synaptic transmission in the ACC can directly contribute to chronic pain itself. Second, ACC neurons may affect spinal pain transmission by its descending facilitatory modulation [8,36]. Indeed, many deeper layer ACC pyramidal cells are projecting cells. In a recent study, we have demonstrated that ACC layer V pyramidal cells are projected to the dorsal horn of the spinal cord [15]. In this study, we provide further evidence that postsynaptic AMPAR GluA1 is increased in spinal projecting ACC neurons. It is likely that enhanced excitatory transmission of the spinal projecting ACC neurons may contribute to increased descending facilitatory modulation in chronic pain conditions [2,8,36]. Our studies provide strong evidence that inhibiting accumulated GluA1 in the deep layers of ACC may inhibit sensory pain transmission at the dorsal horn of the spinal cord. Future *in vivo* electrophysiological experiments are needed to confirm this descending modulation of spinal sensory synaptic transmission.

Our previous studies using pharmacological and genetic approaches consistently demonstrate that GluA1 is critical for ACC LTP [8,16,17]. In the present study, by taking advantage of the SDS-FRL method, we observed the morphological characteristic of the postsynaptic area and confirmed that nerve injury induced GluA1 accumulation in the layer V of the ACC. After peripheral nerve injury, postsynaptic accumulation of GluA1 receptors mediates the LTP of the excitatory synaptic transmission in the SC projecting ACC neurons. Such potentiation of corticospinal projections may contribute to the prolonging of a chronic pain condition. Thus, selective inhibition of the potentiated ACC-SC neurons may cause a direct analgesic effect. Furthermore, considering that long term potentiated synaptic transmission could be observed in the ACC after nerve injury and TBS induced LTP is occluded in animal model of neuropathic pain [15,30], our results also provide possible explanation for the occlusion of LTP in chronic pain conditions: the recruitment of GluA1 into synapses prevents any further AMPAR insertion triggered by LTP induction protocol.

In addition to *in vitro* electrophysiological experiments in the present study and in previous works [15], we performed pharmacological experiments to determine if inhibition of ACC LTP by microinjection of NASPM into the ACC produced inhibitory effects in mechanical hyperalgesia caused by nerve injury. As expected, bilateral microinjections of NASPM into the ACC produced significant analgesic effects in adult mice with nerve injury. This finding provides a novel strategy for chronic pain treatment - inhibiting the potentiated activity of corticospinal projecting neurons may produce a direct analgesic effect. Previous studies revealed the descending pain facilitation system may rely on brainstem rostral ventromedial medulla (RVM) cells, and serotonergic projection from RVM-spinal cord is likely play key roles for the descending facilitation [37-40]. The present study provides a new cortical-spinal projecting pathway that may not require the involvement of RVM. Our preliminary *in vivo* whole-cell patch-clamp studies show that stimulation of the ACC directly potentiated the activity of spinal cord neurons (Chen et al., unpublished data). Considering glutamate is the major transmitter for most of the pyramidal cells in the ACC, it also raises the possibility that glutamate may act as a transmitter for descending pain modulation in the spinal cord. Different types of glutamate receptors in the spinal cord, such as NMDA, AMPA, kainate and metabotropic glutamate receptors may acts as possible candidates for the amplified excitation in the spinal cord [41-43]. Future studies are needed to reveal the exact synaptic mechanisms.

In a previous study, we observed, through western blot analysis, that nerve injury increases phosphorylated-GluA1 expression in layer II/III neuronal membrane in the ACC [11]. However, it is unknown if this PKA phosphorylation is actually required for behavioral hyperalgesia. Through the use of mice with PKC or PKA phosphorylation site mutations, we showed that PKA phosphorylated site ser-845 but not PKC phosphorylated site ser-831 on GluA1 is necessary for neuropathic pain processes. This finding is consistent with previous works in the ACC that show the requirement of adenylyl cyclase type 1 (AC1)-cAMP signaling pathway for the induction of ACC LTP: ACC LTP is blocked in gene knockout mice lacking AC1 [44], and a selective AC1 inhibitor NB001 prevented the induction of LTP [45]. In addition, we have to point out that the mutant mice we used are not regionally specific for ACC. Thus, we cannot rule out the possible compensation or contribution of other brain areas to behavioral efforts we found. However, the analgesic effect that arises from PKA inhibition in the ACC can, at least partially, confirm the importance of PKA phosphorylation for the development of mechanical hyperalgesia.

The present study provides strong evidence for the LTP effect in layer V pyramidal cells in the ACC in chronic neuropathic pain conditions. At the synaptic level, this is the first study to show that AMPARs GluA1 postsynaptic accumulation mediates the nerve injury-induced LTP in the ACC output neurons, especially those projecting to the spinal cord. At circuit level, our results suggest that potentiated synaptic responses may increase intrinsic excitability of the corticospinal projecting neurons and subsequently trigger spinal facilitation. In case of nerve injury, this ACC-spinal cord loop is activated and contributes to the maintenance of hyperalgesia. These findings thus provide new methods and targets for chronic pain treatment. One may reduce chronic pain by inhibiting injury triggered potentiation in the cortex and/or inhibiting descending facilitation by corticospinal projections from the ACC. Future studies are clearly needed to identify the transmitters and mechanisms for such cortico-spinal descending facilitation in different chronic pain conditions.

## Methods

### Animals

Adult male C57BL/6, GluA1 serine-831 and serine-845 phosphorylation site mutant (s831A and s845A) mice were used. Animals were randomly housed under a 12-h light–dark cycle (9 a.m. to 9 p.m. light), with food and water freely available, at least one week before carrying out experiments. All procedures involving animals were under the guidelines of the Fourth Military Medical University, Xi’an Jiaotong University, University of Toronto, Johns Hopkins University and National Institute for Physiological Sciences Animal Care and Use Committee.

### Nerve injury model

A model of neuropathic pain was induced by the ligation of the common peroneal nerve (CPN) as described previously [11,30,46]. Briefly, mice were anesthetized by an intraperitoneal injection of a mixture saline of ketamine (0.16 mg/kg) and xylazine (0.01 mg/kg). The CPN was visible between the anterior and posterior groups of muscles, running almost transversely. The left CPN was slowly ligated with chromic gut suture 5–0 until contraction of the dorsiflexor of the foot was visible as twitching of the digits. The skin was then sutured and cleaned. Sham surgery was conducted in the same manner, but the nerve was not ligated. The mice were used for behavior and/or electrophysiological studies on postsurgical days 7.

### Cannulation and microinjection

Cannulation and microinjection were performed as described previously [30]. After anesthesia, the head of the mouse was fixed on a stereotaxic frame and the scalp was shaved and then cleaned with povidone-iodine and alcohol. An incision was made over the skull and the surface was exposed. Two small holes were drilled above the ACC and guide cannulas were placed so that the final coordinates for microinjection would be 0.7 mm anterior to the bregma, 0.3 mm lateral to the midline, and 1.75 mm ventral to the surface of the skull for the ACC, according to the atlas of Paxinos and Watson (1999). For microinjection, the mice were restrained in a plastic cone and a small hole over the guide cannulas was made. A 30-gauge injector 0.7 mm lower than the guide was used. The microinjection was conducted using a motorized syringe pump (Razel Scientific Instruments, CT, USA) and a Hamilton microsyringe (Hamilton, Reno, NY, USA). Pure saline (0.9%), NASPM (5 μM), AP5 (50 μM) or Rp-cAMP (2 μM) dissolved in saline was delivered bilaterally into the ACC (0.5 μl/side, total 1 μl). The injector was left in place for 1 min.

### Behavior observation

One week following nerve ligation or sham surgery, mice paw withdrawal threshold was tested with *von Frey* filaments applied to the paw [47]. The animals were placed in Lucite cubicles over a wire mesh and acclimated for 30 min before testing. A series of filaments (0.008, 0.02, 0.04, 0.16, 0.4, 0.6, 1, 1.4, 2 g) with various bending forces (according to 0.078, 0.196, 0.392, 1.568, 3.92, 5.88, 9.8, 13.72, 19.6 mN) were applied to the plantar surface of the hindpaw ipsilateral to the nerve injury side until the mice withdrew from the stimulus. Each filament was applied twice. The lowest force at which a withdrawal response was obtained was taken as the paw withdrawal threshold.

### SDS digested freeze-fracture replica labeling

Animals were perfused with 0.1 M PB (pH 7.4) which contained 2% paraformaldehyde and 15% picric acid. Blocks containing the ACC were cut into 150 μm-thick sections with a microslicer (Leica Microsystems VT1000s). After ACC layer V regions were trimmed out (about 0.5 mm^2^), they were cryprotected with 10%, 20% glycerol in 0.1 M PB for 30 min, and 30% glycerol in 0.1 M PB overnight at 4°C and then frozen with a high-pressure freezing machine (HPM 010; Bal-Tec, Balzers, Liechtenstein). The frozen sections were fractured in a freeze-etching device (BAF 060; Bal-Tec) and the fractured faces were first replicated by deposition of carbon (5 nm, rotating) with an electron beam gun positioned at a 45^0^ angle, shadowing unidirectionally by platinum (2 nm) with the gun positioned at a 60^0^ angle, followed by carbon (15 nm) evaporated from a 45^0^ angle. Tissue debris attached to replicas were dissolved with a solution containing 2.5% SDS and 20% sucrose made up in 15 mM Tris buffer (pH 8.3) under gentle shaking at 80°C for 18 hours. Replicas were then washed in 25 mM TBS containing 0.05% BSA three times and incubated in a blocking solution containing 5% BSA in 25 mM TBS for 1 h. Subsequently, the replicas were incubated in a purified anti-c terminal of GluA1 antibody (1:1000, kindly provided by Dr. Makoto Itakura diluted in 25 mM TBS containing 1% BSA for 36 hours at 4°C. After several washes, the replicas were incubated in goat anti-rabbit IgG coupled to 5 nm gold particles (1:30; BioCell Research Laboratories) diluted in 25 mM TBS containing 5% BSA overnight at 4°C. After washing in 25 mM TBS containing 0.05% BSA three times and double distilled water once, the replicas were picked up on 100-line copper grids and analyzed under a Tecnai12 electron microscope (Philips, Netherlands).

Clusters of intramembrane particles (IMP) on the exoplasmic fracture-face (E-face) were taken as excitatory postsynaptic specialization [48]. IMP clusters were defined as packed IMPs with less than 15 nm distance from each other. Here, we considered an IMP cluster as postsynaptic membrane specialization if the cluster contained at least 30 IMPs. Immunogold particles were regarded as associated with the postsynaptic membrane specialization if they were above or in the immediate vicinity (not further than 20 nm from the edge) of the IMP cluster. The outline of synaptic sites was demarcated freehand, and areas of IMP clusters were measured with Image J software. Immunoparticles within the demarcated IMP cluster were counted manually. The density of immunoparticles for GluA1 was calculated by dividing the number of immunoparticles by the area of IMP cluster.

### HRP retrograde labeling and pre-embedding EM methods

The procedure for retrograde tracer WGA-HRP injection into the spinal cord (SC) or ventral striatum (VS) was according to our previous works [49,50]. The anesthetic mice were fixed on a stereotaxic frame. For the SC injection, the skin between scapulas was incised and paravertebral muscles were cut off and vertebral plate of the fourth cervical vertebra was exposed. The vertebral plate was removed and the intumescentia cervialis was exposed. For VS injection, the skull was exposed, and a hole was drilled through the skull over the VS (0.38 mm anterior to bregma, 2.0 mm lateral to the midline and 4.5 mm ventral to the surface of the skull for the VS). Then 0.4 μl WGA-HRP (40 mg/ml, Vector Laboratories, Burlingame, U.S.A.) was injected into one side of the SC or VS. Brain samples were sliced for pre-embedding immunostainning after 72 hrs. Sections were processed for the histochemical demonstration of WGA-HRP by using the tetramethy-lbenzidine-sodium tungstate (TMB-ST) method and the HRP reaction products were intensified with DAB/Cobalt/H_2_O_2_ solution. After histochemical confirmation of HRP reactive product, the sections were washed several times in 0.1 M PB. Sections were cryoprotected in solutions containing a mixture of 30% (w/v) sucrose and 10% (v/v) glycerol in 0.05 M PB for 30 min. The sections were freeze-thawed with liquid nitrogen and then incubated in a blocking solution containing 20% normal goat serum (NGS) in 0.05 M Tris-buffered saline (TBS; pH 7.4) for 30 min to block the non-specific immunoreactivity, followed by incubation with rabbit anti-GluA1 antibody (1:600) at 4°C for 12 hrs. After washing in TBS, the sections were incubated for 8 hours in 1:100-diluted anti-rabbit antibody conjugated to 1.4 nm gold particles (Nanoprobes; Stony Brook, NY, U.S.A). The sections were then processed as follows: (1) 1% postfixation with glutaraldehyde in 0.1 M PB for 10 min; (2) silver enhancement with an HQ Silver Kit (Nanoprobes, Stony Brook, NY, U.S.A). The sections were then treated with 1% OsO_4_ in 0.1 M PB for 1 hr. Subsequently, the sections were counterstained with 1% (w/v) uranyl acetate in 70% ethanol for 1 hr. After dehydration, the sections were mounted on silicon-coated glass slides and flat embedded in epoxy resin (Durcupan; Fluka, Buchs, Switzerland). Under electron microscope, HRP-labeled neurons were detected by the presence of highly electron-dense clumps of crystalline material and sometimes by amorphous punctual structures in the cytoplasm of the dendrites and somata. GluA1 immunoreactivity was determined by the presence of the immunogold-silver particles which were distributed in the cytoplasm of the somata and dendrites. Asymmetric synaptic junction containing more than 3 immunogold-silver grain particles were considered as GluA1-immu-noreactive postsynaptic structure.

### Extracellular field EPSP recording

The MED64 probe (P515A, Panasonic, Japan) has an array of 64 planar microelectrodes, arranged in an 8 × 8 pattern, with an interpolar distance of 150 μm. Before use, the surface of the MED64 probe was treated with 0.1% polyethyleneimine (Sigma, St. Louis, MO; P-3143) in 25 mmol/L borate buffer (pH 8.4) overnight at room temperature. Then the probe surface was rinsed three times with sterile distilled water [32,33]. Acute coronal brain slices (300 μm) containing the ACC, were prepared using standard methods [11,30,51]. After 1 hr for recovery in standard ACSF, slices were transferred to the recording chamber and subfused with ACSF at 28-30°C and maintained at a 2 ml/min flow rate. The slices were positioned on the MED64 probe in such a way that the middle part of the probe close to the central point of the ACC. One of the channels located in the superficial layers (II-III) of the ACC, from which the best synaptic responses can be induced in the surrounding channels in deep layers (V), was chosen as the stimulation site.

Slices were kept in the recording chamber for at least 1 hr before the start of experiments. Bipolar constant current pulse stimulation (1–10 μA, 0.2 ms) was applied to the stimulation channel and the intensity was adjusted so that a half-maximal field excitatory postsynaptic potential (fEPSP) was elicited in the channels closest to the stimulation site. The fEPSP responses were sampled every 1 min and averaged every 4 min. The parameter of “slope” indicated the averaged slope of each fEPSP recorded by activated channels. Stable baseline responses were first recorded until the baseline response variation is less than 5% in most of the active channels within 1 hr. In some cases, a theta-burst stimulation (TBS, five trains of bursts with four pulses at 100 Hz, at 200 ms interval; repeated five times at intervals of 10 s) was applied to the stimulation channel to induce L-LTP.

### Whole-cell patch-clamp recordings on retrograde labeling ACC neurons

0.25% DiI distilled in saline solution was unilaterally pressure-injected (1 μl) into the C4-5 SC (in the same time with CPN ligation or sham surgery) or VS (4 days after CPN ligation or sham surgery) with a Hamilton microsyringe attached with a glass micropipette (tip outer diameters ranged from 10–20 μm). Those mice were allowed to survive for 3–7 days before whole cell patch experimental procedures. Then mice were sacrificed and coronal brain slices (300 μm) at the level of the ACC were prepared using standard methods. Slices were transferred to a submerged recovery chamber containing oxygenated (95% O_2_ and 5% CO_2_) ACSF (124 mM NaCl, 4.4 mM KCl, 2 mM CaCl_2_, 1 mM MgSO_4_, 25 mM NaHCO_3_, 1 mM NaH_2_PO_4_, and 10 mM glucose) at room tempe-rature for at least 1 hr and then heated up to 32°C for recording. Evoked EPSCs were recorded from layer V neurons in randomly selected sides of the ACC, with an Axon 200B amplifier, and stimulations were delivered by a bipolar tungsten stimulating electrode placed in layer II/III of the ACC. AMPAR-mediated EPSCs were induced by repetitive stimulations at 0.02 Hz, and neurons were voltage-clamped at −60 mV in the presence of AP5 (50 μM) and picrotoxin (100 μM). The recording pipettes (3–5 MΩ) were filled with a solution containing (in mM) 112 Cs-Gluconate, 5 TEA-Cl, 3.7 NaCl, 0.2 EGTA, 10 HEPES, 2 MgATP, 0.3 Na_3_GTP and 5 QX-314 (adjusted to PH 7.2 with CsOH, 290 mOsmol). The initial access resistance was 15–30 MΩ, and it was monitored throughout the experiment. Data were discarded if the access resistance changed >15% during experiment. Data were filtered at 1 kHz, and digitized at 10 kHz.

Retrograde labeling cells were observed under FV-1000 confocal microscope under proper filters for DiI (excitation 549 nm; emission 565 nm). In some cases, biocytin (0.5%) was distilled into the recording pipette for subsequent observation of the patched neurons. After recording, the slices with biocytin labeled neurons were fixed with 4% paraformaldehyde in 0.1 M PB (pH 7.4) for 2 hrs at room temperature. Then the slices were immunostained with Alexa-594 conjugated avidin (1:200), thoroughly washed and observed under FV-1000 confocal microscope for DiI and Alexa-594 labeled neurons by using standard method.

### NSFA

Nonstationary peak-scaled fluctuation analysis (NSFA) [30,52] was performed on the AMPAR mediated eEPSC using Mini Analysis Program 6.0.3. The eEPSCs were recorded on ACC-SC or ACC-VS projecting neurons in layer V of the ACC and induced by applying a constant intensity (8 V) in layer II/III. After a stable baseline recording for 10 min, NASPM was bath applied for another 20 min and data were collected for analysis. When doing NSFA, the eEPSCs were aligned on the rising phase at the half-maximal amplitude and averaged. The variance of the individual trace *v. s*. the averaged trace at decay phase was calculated and plotted against the bin amplitude of the averaged response. The collected data were fitted with the following parabolic function using a least-squares algorithm:

$$ {\sigma}^2=iI-\frac{I^2}{N_p}+{\displaystyle {\sigma}_b^2} $$

Where the *N*_*p*_ was the number of active channels, which was defined as number of physical channels × open probability. *I* is the bin value, *σ*^*2*^ is the variance, *σ*_*b*_^*2*^ indicates the variances of background.

The unit conductance was calculated as:

$$ \upgamma =\mathrm{i}/\left({\mathrm{V}}_{\mathrm{h}}\hbox{-} {\mathrm{V}}_{\mathrm{rev}}\right) $$

*V*_*h*_ indicates the holding potential, *V*_*rev*_ indicated the reversal membrane potential of the eEPSC (about 0 mV) [53].

### Statistical analyses

All experiments were carried out as blind to genotype and the conditions of the experiments, unless indicated in naïve animals. Data were collected and processed randomly, and no data points were excluded. No statistical methods were used to predetermine sample sizes, but our sample sizes were similar to those reported in previous publications. Statistical comparisons were made using the unpaired, paired *t*-test or two-way ANOVA (Tukey test was used for *post hoc* comparison). The normal distribution and the variation within each group of data were verified by using Sigmaplot 11.0 software before applying statistical comparison. Analyzed numbers (n) for each set of experiments are indicated in the corresponding figure legends or main text sections. The examples shown in each figure are representative and were reproducible at least three times for each set of experiments. All data were presented as the Mean ± S.E.M. In all cases, *p* <0.05 was considered statistically significant.

## Abbreviations

AC1: Adenylyl cyclase type 1
ACC: Anterior cingulate cortex
ACSF: Artificial cerebrospinal fluid
CPN: Common peroneal nerve
EM: Electron microscopic
EPSC: Excitatory postsynaptic current
FRL: Freeze-fracture replica labeling
HRP: Horseradish peroxidase
IMP: Intramembrane particle
LTP: Long term potentiation
LTD: Long term depression
NSFA: Nonstationary fluctuation analysis
PKA: Protein kinase A
PKC: Protein kinase C
RVM: Rostral ventromedial medulla
SC: Spinal cord
TBS: Theta-burst stimulation
VS: Ventral striatum.
